# Resistant Starch Type 5 Formation by High Amylopectin Starch–Lipid Interaction

**DOI:** 10.3390/foods13233888

**Published:** 2024-12-02

**Authors:** Fernanda G. Castro-Campos, Edgar A. Esquivel-Fajardo, Eduardo Morales-Sánchez, Mario E. Rodríguez-García, Oscar Yael Barron-Garcia, Cristian Felipe Ramirez-Gutierrez, Guadalupe Loarca-Piña, Marcela Gaytán-Martínez

**Affiliations:** 1 Posgrado en Ciencia y Tecnología de los Alimentos, Facultad de Química, Universidad Autónoma de Querétaro, Centro Universitario, Santiago de Querétaro 76010, Mexico; fer.castro207@gmail.com (F.G.C.-C.); ed.esquivel.f@gmail.com (E.A.E.-F.); loarca@uaq.mx (G.L.-P.); 2 Instituto Politécnico Nacional, CICATA-IPN Unidad Querétaro, Cerro Blanco No. 141, Col. Colinas del Cimatario, Santiago de Querétaro 76090, Mexico; emoraless@ipn.mx; 3 Centro de Física Aplicada y Tecnología Avanzada, Departamento de Nanotecnología, Universidad Nacional Autónoma de México, Campus Juriquilla, Querétaro 76230, Mexico; marioga@fata.unam.mx (M.E.R.-G.); yaelbarron@fata.unam.mx (O.Y.B.-G.); 4 División Industrial, Universidad Tecnológica de Querétaro, Av. Pie de la Cuesta 2501, Nacional, Santiago de Querétaro 76148, Mexico; 5 Cuerpo Académico de Tecnologías de la Información y Comunicación Aplicada, Universidad Politécnica de Querétaro, El Marqués 76240, Mexico; cristian.ramirez@upq.edu.mx

**Keywords:** amylopectin–lipid complex, orthorhombic, oleic acid, waxy starch, gelatinization

## Abstract

The formation of resistant starch type 5 (RS5), primarily associated with amylose–lipid complexes, is typically attributed to starches with high-amylose content due to their affinity for lipid interactions. Recently, studies have also investigated the potential of amylopectin-rich starches to form amylopectin–lipid complexes (ALCs), expanding RS5 sources. This study explores the capacity of waxy corn starch (WS), which is rich in amylopectin, to develop ALCs with oleic acid (10% *w*/*w*) under different thermal and mechanical conditions. Specifically, WS was treated at temperatures of 80 °C, 85 °C, and boiling, with stirring times of 0 and 45 min. Results demonstrated significant ALC formation, reaching a peak complexation index (CI) of 59% under boiling conditions with 45 min of stirring. Differential scanning calorimetry (DSC) identified a notable endothermic transition at 110 °C, indicating strong ALC interactions. FTIR spectra further evidenced starch–lipid interactions through bands at 2970 cm^−1^ and 2888 cm^−1^. X-ray diffraction (XRD) analysis confirmed the presence of orthorhombic nanocrystals in native WS, with ALC samples exhibiting a V-type diffraction pattern, supporting effective complexation. This study advances knowledge on starch–lipid interactions, suggesting ALCs as a promising RS5 form with potential food industry applications due to its structural resilience and associated health benefits.

## 1. Introduction

Resistant starch type 5 (RS5) is highly valued in the food industry for its versatility and functional properties [1]. Its popularity stems from its accessibility, cost-effectiveness, non-toxicity, biodegradability, and ability to selectively release bioactive compounds within the gastrointestinal tract, making it an excellent candidate for targeted nutritional applications [2]. Consequently, exploring cost-effective and efficient methods to produce this type of resistant starch is essential, particularly those that can also deliver enhanced health benefits. Amylose–lipid complexes, also known as RS5, are formed through interactions between fatty acids and the amylose helix. However, several factors significantly influence the formation of these complexes [3,4,5]. These factors include the amylose content of the starch, the type of lipid involved in the interaction, and the processing method used [6,7,8,9,10,11]. Understanding these factors is crucial for comprehending how RS5 is formed and characterized during the various processes used to obtain it.

Starches with a high amylose content have been shown to produce a greater proportion of RS5 because amylose molecules exhibit a strong affinity for interacting with lipid compounds, leading to the formation of amylose–lipid complexes [12,13,14,15]. This is primarily due to the hydrophobic properties of the amylose helix, which enable it to interact and self-assemble with other amylose helices and non-polar lipids [16,17,18]. However, obtaining such starches can often involve genetic modification, which may make them a costly source of raw material [19]. Moreover, when analyzing interactions with other macromolecules and the effects of various processing methods, it is essential to recognize that starch should be considered a complex particle. In this context, the formation of amylose–lipid complexes particularly depend on the ability of gelatinized amylose to align into a single helix around the complex-forming element, a process facilitated by interactions between methylene groups and glycosidic bonds [20]. While the physical properties of amylose are widely acknowledged as crucial for the formation of amylose–lipid complexes, the temperatures typically used are often insufficient to achieve gelatinization of amylose-rich starches [21]. In this context, the parameters governing gelatinization are critical for the successful formation of RS5.

Regarding temperature, it has been reported that the formation of starch–lipid complexes depends on the size of the interacting macromolecule. The formation of RS5 is possible through thermal treatments at temperatures above 100 °C. This suggests that complex formation occurs during the gelatinization of starch; as such, temperatures allow the opening of the double helix structure of amylose chains or the fragmentation of amylopectin molecules. Therefore, starches rich in amylopectin may be a more accessible source for RS5 formation. In this context, the formation of these complexes using high-amylopectin rice starch through enzymatic debranching has been reported [22,23,24]. This process results in fractions with shorter chain lengths, and the studies highlight that these shorter chains can facilitate interactions with lipids. In another study, Luo et al. [25] reported that the size of the chains obtained by amylopectin debranching significantly affects their ability to interact with lipids. Specifically, wider helical fragments consisting of eight glucose units per turn were significantly more prone to form complexes with free fatty acids than shorter, narrower chains with fewer than eight glucose residues per turn. However, the enzymatic pathway for RS5 production is often costly and yields relatively low amounts, making it less attractive for industrial applications. An alternative approach could involve modifying amylose and amylopectin chains through thermal treatments, providing a more practical option [26,27]. The above suggests that an interaction between fatty acid and amylopectin-rich starch under specific conditions could result in an increased RS5 content. This opens new possibilities for creating RS5 from various starch sources, not limited to those high in amylose.

On the other hand, the type of fatty acid influences the formation of complexes with starch molecules and affects the content of RS5. Saturated fatty acids have been found to promote the formation of these complexes more effectively than unsaturated fatty acids, resulting in higher levels of RS5. Ai et al. [28] studied the effects of various types of lipids, including triglycerides (e.g., corn oil), phospholipids (e.g., soy lecithin), and free fatty acids with different chain lengths and degrees of unsaturation, on the enzymatic hydrolysis and thermal properties of starches from different sources such as normal corn, tapioca, waxy corn, and high-amylose corn. The authors reported that the type of lipid used significantly influenced the enzymatic hydrolysis rate and the thermal properties of the starch. Specifically, complexes formed with oleic acid exhibited lower degrees of hydrolysis. Oleic acid, a monounsaturated fatty acid, is known for its health benefits, including potential positive effects on cardiovascular health, could therefore enhance the nutritional value of resistant starch. In this context, the formation of resistant starch with essential fatty acids like oleic acid could confer additional health benefits [28,29,30,31].

These findings suggest a possible interaction between oleic acid and amylopectin-rich (waxy) starch under certain conditions, leading to a higher content of RS5 [26,32]. Understanding the amylopectin–lipid interaction could potentially lead to the development of new functional starches and ingredients for the food industry, offering potential applications in addition to health benefits.

Other studies have looked at the gelatinization process and the involvement of starch components in a physical way. According to Vega-Rojas et al. [33] and Esquivel-Fajardo et al. [34], the gelatinization of starch involves the solvation of the orthorhombic and/or hexagonal crystal structures. This solvation process requires a suitable temperature, water concentration, and exposure time. Therefore, the formation of RS5 can be influenced by the processing conditions, including time and temperature during the gelatinization. However, further studies are needed to fully understand the interactions between the main components of starch, and the mechanisms behind the formation of amylose–lipid and amylopectin–lipid complexes, as well as the possible presence of nanocrystals.

Therefore, the aim of this study was to investigate the formation of amylopectin–lipid complexes using high-amylopectin maize starch (waxy) to produce RS5. Oleic acid was employed as the complexing lipid under different conditions, including temperatures of 80, 85 °C, and boiling, and stirring times of 0 and 45 min. The interaction between amylopectin and oleic acid in forming RS5 was thoroughly evaluated using structural characterization techniques, including X-ray diffraction and transmission electron microscopy (TEM), scanning electron microscopy (SEM) for morphological analysis, FTIR vibrational spectroscopy, and resistant starch quantification.

## 2. Materials and Methods

### 2.1. Biological Material Results

In this study, a commercially available high amylopectin (waxy) corn starch (Amioca, CODE: 04401106) from Ingredion ^®^ (Ingredion Mexico, S.A. de C.V., Queretaro, Qro City, Mexico) was used. The waxy corn starch contained 10.0 ± 0.3% moisture, 1.1 ± 0.2% ash, 0.2 ± 0.2% lipids, 0.3 ± 0.1% protein, 98.4 ± 1.2% total starch, 11.1± 0.3% amylose, 87.3 ± 0.3% amylopectin on a dry matter basis. Oleic acid (O1008 Sigma Aldrich^®^, St. Louis, MO, USA) was used as the test lipid.

### 2.2. Amylopectin–Lipid Complex Formation

To obtain the amylopectin–lipid complexes, 20 g of waxy corn starch was stirred in 30 mL of distilled water at room temperature. Separately, 220 mL of water was heated to 80, 85 °C, and boiling temperature. The previously hydrated starch was then added to the heated water. Once the mixture reached the desired temperature, oleic acid was added at a concentration of 10% (*w*/*w*), having been previously dissolved in 10 mL of ethanol.

**Figure 1:**
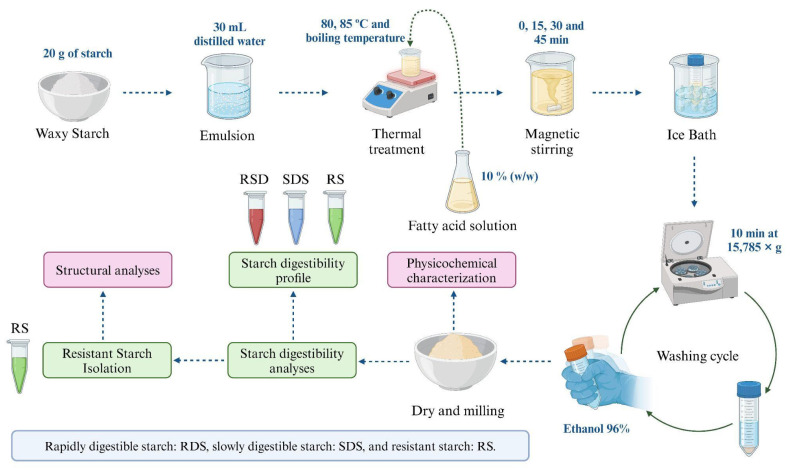
Flow diagram describing the production of amylopectin–lipid complexes.

The mixture of starch, water, and fatty acids was stirred continuously for 0, 15, 30, and 45 min for each treatment. After stirring, each mixture was cooled in an ice bath for 10 min. The samples were then centrifuged at 15,785× *g* for 10 min to remove excess water. The resulting precipitate was washed with ethanol to remove the free fatty acids. Finally, the samples were dried at 40 °C for 24 h, ground, sieved through a 60-mesh screen, and stored in plastic bags for further analysis (Figure 1).

### 2.3. Complexation Index

The complexation index (CI) was measured according to the method described by Chao et al. [12]. The amylopectin–lipid complex (0.4 g) was transferred into 50 mL tubes and diluted with water to a total weight of 5 g. The CI was determined as follows: The suspension was boiled in a water bath for 10 min with constant stirring. After heating, each sample was cooled at room temperature (25 ± 1 °C), then 25 mL of distilled water was added, and the sample was shaken for 2 min. The sample was then centrifuged at 3000× *g* for 15 min. A 500 μL portion of the supernatant was transferred to a test tube, followed by the addition of 15 mL of distilled water and 2 mL of iodine solution (2.0% KI and 1.3% I_2_ in distilled water). The absorbance at 620 nm was measured using a UV-VIS spectrophotometer (Thermo Scientific, Waltham, MA, USA. Multiskan Ascent, model 51118307). Starch without added lipids was used as a reference. Finally, the CI was calculated using the following equation (Equation (1)):

(1) $$\%CI=\left(\frac{{Abs}_{reference}-{Abs}_{starch-lipid}}{{Abs}_{reference}}\right)\times 100,$$

where *Abs_reference_* is the absorbance of the reference starch solution without treatment, and *Abs_starch-lipid_* is the absorbance of the starch–lipid mixture.

### 2.4. Scanning Electron Microscopy Analysis

The morphology of native waxy corn starch, amylopectin–oleic acid complexes, and isolated RS5 was observed using a scanning electron microscope (JEOL, JSM-6060LV, Kenji Kazato and Kazuo Ito in Mitaka, Tokyo) with an accelerating voltage of 5 kV and a secondary electron detector. Samples were mounted on a sample holder with carbon tape and gold-coated prior to analysis [7].

### 2.5. Nanocrystals Isolation

For the isolation of nanocrystals from waxy corn starch, the method proposed by Rojas-Molina et al. [35] was employed. Specifically, 37 g of starch was suspended in 260 mL of sulfuric acid (H_2_SO_4_, 3.16 M). The solution was maintained at 40 °C for 7 days with constant stirring. The crystalline fraction of starch was recovered from the solution by centrifugation at 15,785× *g*. Subsequently, the resulting pellet was washed with distilled water until a pH of 5 was reached. Finally, to prevent solvation of the nanocrystals, an ammonia treatment was performed to adjust the pH to 8–8.5, and the sample was stored at 4 °C.

### 2.6. Transmission Electronic Microscopy (TEM)

The aim of acquiring transmission electron microscopy (TEM) images of nanocrystals was to determine the crystal directions and correlate them with the powder diffraction data reported by Rodriguez-Garcia et al. [36] to ascertain the crystal structure.

TEM images of the isolated nanocrystals (INs) isolated from waxy corn starch were obtained using a JEOL JEM-2200 FS transmission electron microscope (TEM) according to the method proposed by Rojas-Molina et al. [35]. The sample was ultrasonically dispersed in isopropyl alcohol and deposited onto a 3 mm copper grid using a capillary tube. The gird was then placed in a plasma cleaner (Glow Discharge Plasma Cleaner, SKU: M-PDC-32G9, NY, USA) to remove volatile organics before imagining. TEM measurements were conducted at an accelerating voltage of 80 kV. The crystal size of the nanocrystal was determined using the Digital Micrograph V 4.0 software (Gatan Inc. Pleasanton, CA, USA). An array mask was applied to clean the FFT signal and calculate the interplanar distances.

### 2.7. X-Ray Diffraction Patterns

X-ray diffraction patterns of the waxy corn starch amylopectin–lipid complex and the isolated RS were obtained using a Rigaku diffractometer (Ultima IV model, Cedar Park, TX, USA) equipped with a D/tex Ultra detector at 35 Kv and 15 mA. CuKα radiation (λ = 1.5405 Å) was used, scanning the sample from 5 to 35 ° (2θ scale) with a step of 0.02° [36].

### 2.8. Differential Scanning Calorimeter Analysis

The waxy corn starch sample and the amylopectin–lipid complex were analyzed using a differential scanning calorimeter (DSC 1 Star^®^ System, Mettler Toledo, Greifensee, Switzerland). The thermograms were obtained according to the method published by Cervantes-Ramírez et al. [6], with slight modifications. Briefly, 0.5 g of each sample was mixed with the distilled water to achieve 60% moisture content on a dry basis. The mixtures were then stabilized at room temperature in sealed bags for 30 min. Subsequently, 10 ± 0.1 mg of each stabilized sample was weighed into an aluminum pan (40 µL), and the pan was sealed with the T-press instrument. The sample was heated from 30 to 150 °C at 10 °C/min. These procedures were performed in duplicate.

### 2.9. Vibrational Characterization

The functional groups for the waxy corn starch components and amylopectin–lipid complexes were determined using the method proposed by Cervantes-Ramírez et al. [6] and Cabrera-Ramirez, et al. [7], using a Perkin Elmer IR spectrometer (Spectrum Two model, Waltham, MA, USA) equipped with a diamond-coated attenuated total reflectance (ATR) accessory.

### 2.10. Rapidly Digestible Starch, Slowly Digestible Starch, and Resistant Starch

The in vitro digestibility of starch was determined according to the method described in the literature [37], with some modifications. Starch and the amylopectin–lipid complex (100 mg each) were added to a centrifuge tube containing 12 mL of sodium acetate buffer (pH: 5.2). The mixture was shaken for 20 s. A 0.5 mL aliquot, labeled GF (glucose-free) was then withdrawn. Subsequently, 300 µL of amyloglucosidase and 100 µL of pancreatic amylase were added. After enzyme addition, the mixture was incubated at 37 °C with constant stirring. Aliquots of 0.5 mL were withdrawn after 20, 120, and 180 min. Each aliquot was transferred to another tube, where 4.5 mL of ethanol (99%) was added, and the mixture was centrifuged at 3000× *g* for 10 min. Once the reaction was complete, a portion of the obtained pellet was collected from the samples treated at 80, 85 °C, and boiling temperature with stirring times of 0 and 45 min, to characterize the resistant starch fraction. Free glucose was then measured in all treatments using the glucose-peroxidase kit (GAGO20, Sigma Aldrich). Each sample was analyzed in triplicate, and the proportions of rapidly digestible starch (*RDS*), slowly digestible starch (*SDS*), and resistant starch (*RS*) were calculated using the following equations:

(2) $$\%RSD=\left(\frac{{(G}_{20}-FG)\;}{TS}\right)\times 0.9\times 100,$$

(3) $$\%SDS=\left(\frac{{(G}_{120}-G_{20})\;}{TS}\right)\times 0.9\times 100,$$

(4) $$\%RS=\left(\frac{TS-RSD-SDS)\;}{TS}\right)\times 0.9\times 100,$$

where *FG* represents the free glucose content, *G20* for the released glucose content (mg) after 20 min of incubation, *G120* for the released glucose content (mg) after 120 min, and *TS* (total starch) for the total released glucose (mg) after 180 min. *RS* refers to the resistant starch content.

In order to determine the structural changes suffered by the RS fraction after digestion process, a second test was performed according to the method described above. However, after the enzymatic treatment and before RS quantification, the resulting pellet was recovered and analyzed with RDX for all samples.

### 2.11. Design and Statistical Analysis

Data are presented as mean ± standard deviation of independent experiments performed in duplicate. Statistical analysis was performed using Minitab 16 (Minitab Inc., 2010, State College, PA, USA). Analysis of variance (ANOVA) was performed using Tukey–Kramer’s test to determine significant differences at *p* < 0.05 for multiple variables. Pearson correlation was performed to find out the relationship between the studied variables and to determine the treatment that provided the highest formation of RS5.

## 3. Results and Discussion

### 3.1. Complexation Index (CI)

**Figure 2:**
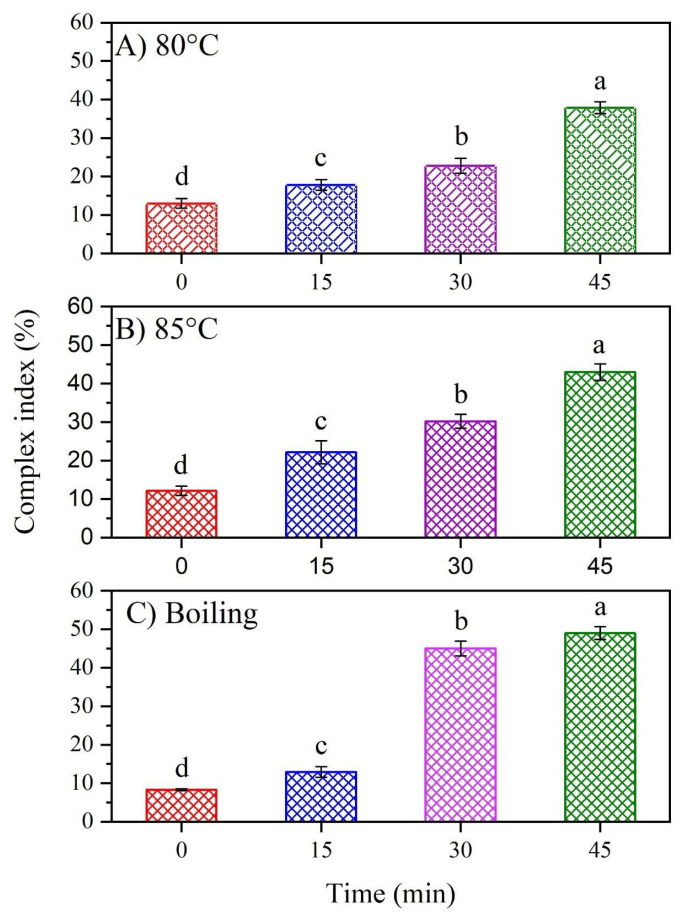
Complexation index of amylopectin and oleic acid at (**A**) 80 °C, (**B**) 85 °C, and (**C**) boiling temperature at different stirring times. Different letters in the same column are statistically different (*p* < 0.05). Table A1 in Appendix A shows the statistical analysis of temperature parameters.

Figure 2 presents the complexation index percentage for each treatment, which indirectly indicates the amylopectin–oleic acid complex formation. Overall, the results show that there is an almost linear relationship between the percentage of complexation index and the increase in temperature and stirring time. Thus, higher temperatures (boiling temperature) and longer stirring times (45 min) result in increased complexation index percentages, indicating greater formation of the waxy starch–oleic acid complex. Significantly higher complex formation was observed at 85 °C (Figure 2B) and boiling temperature (Figure 2C) with a stirring time of 45 min. The observed increase in the complex index can be attributed to the gelatinization of the waxy starch. Previous studies have reported that gelatinization of amylopectin-rich starch occurs between 62 and 73 °C [38]. In this sense, the treatment at 80 °C is within the gelatinization temperature range, suggesting that the waxy starch used in the experiment is partially gelatinized at this temperature. When the temperature increases up to the boiling temperature, the degree of gelatinization of the starch increases further. Consequently, the amylopectin chains are more efficiently released, leading to the opening of the double helix structure of the branching chains and helical opening [39], which increases the complex index (Figure 2C). Furthermore, Figure 2 shows that stirring time significantly influences the complexation index, in addition to the effects of heating temperature. This observation suggests that the duration of stirring, likely impacting the structural arrangement of amylopectin, plays a role as critical as starch gelatinization in enhancing the complexation index.

The observed effects of temperature and stirring time suggest that these parameters play a crucial role in the interaction between amylopectin and oleic acid, facilitating the incorporation of the fatty acid and the formation of the amylopectin–oleic acid complex [40,41]. This interaction may result in the physical entrapment of oleic acid in the amylopectin chains, leading to the formation of a self-assembles complex [16]. The results also indicate that hydrolysis of amylopectin (major component of starch) significantly impacts the proportion of starch that can interact with a lipid chain to form a complex. This hydrolysis can be induced by mechanical agitation such as constant stirring.

Constant stirring leads to a more substantial mechanical breakage of the amylopectin and amylose molecules, resulting in smaller fractions whose helical structures are easier to dissociate. Previous studies have reported that the helical structure of these components can be opened or swelled at certain temperatures, allowing the incorporation of lipids [39]. Moreover, the helical opening of amylopectin plays a crucial role in complexes formation [25]. Accordingly, the present study shows that increased stirring time and temperature enhance the interaction between amylopectin and oleic acid due to the partial hydrolysis of amylopectin.

This study demonstrates the importance of controlling temperature and interaction time between fatty acids and the amylopectin or amylose components of waxy starch to promote the formation of amylopectin–lipid complexes. The type of fatty acid must also be considered, as its molecular structure can influence its incorporation into amylopectin chains. For instance, oleic acid is an 18-carbon monounsaturated fatty acid whose cis-double bond introduces a link in the hydrocarbon chain, causing chain twisting and steric hindrance that may impede the formation of amylopectin–lipid complexes [17].

### 3.2. Scanning Electron Microscopy Analysis (SEM)

**Figure 3:**
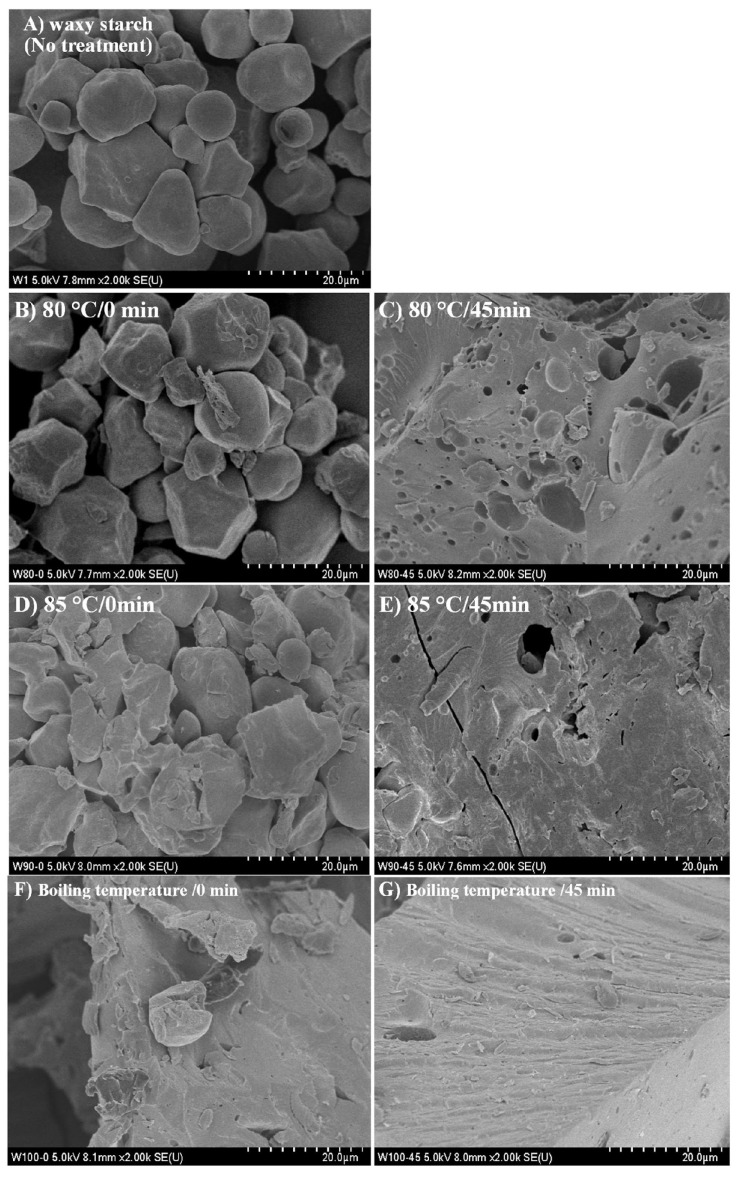

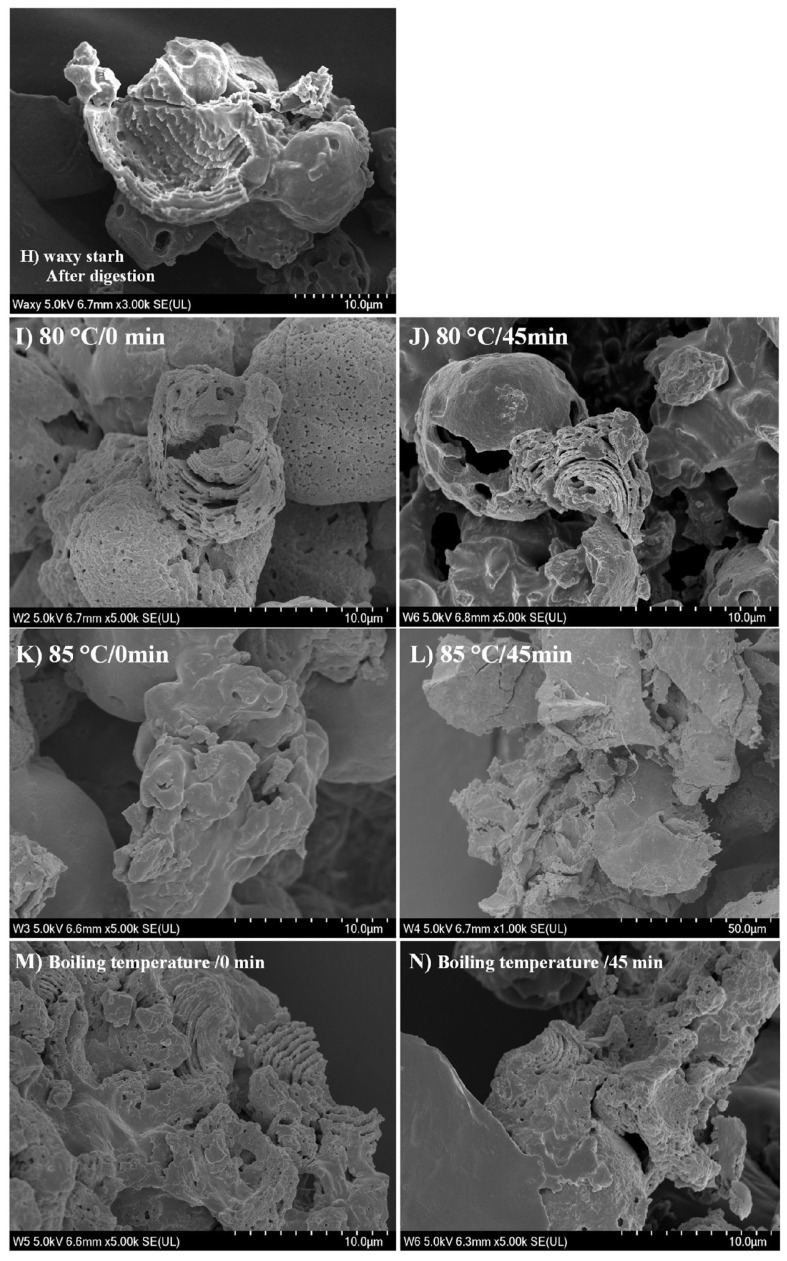
SEM images of the (**A**) native waxy starch and the amylopectin–oleic acid complex at different temperatures and stirring times (**B**) 80 °C at 0 min and (**C**) 45 min; (**D**) 85 °C at 0 min and (**E**) 45 min; and (**F**) boiling temperature at 0 min and (**G**) 45 min. Figure 3 also shows the (**H**) native waxy starch after enzymatic digestion and the amylopectin–oleic acid complex at different temperatures after enzymatic digestion (**I**) 80 °C/0 min, (**J**) 80 °C/45 min, (**K**) 85 °C/0 min, (**L**) 85 °C/45 min, (**M**) boiling temperature/0 min; and (**N**) boiling temperature/45 min.

Figure 3 shows the SEM images used to study the morphological changes of the waxy starch granules at different temperatures and stirring times. Additionally, Figure 3 shows the SEM images of the ALC samples after enzymatic digestion, allowing for the examination of the morphology of the digestion-resistant part.

In its native form (Figure 3A), waxy starch exhibits irregular round and polygonal granules with sizes around 20 µm, which is consistent with the morphology of waxy maize starch granules reported previously [42]. Pores are also visible in some granules, which may be more susceptible to enzymatic attack. This susceptibility is related to the resistant content in their natural state. After the thermal treatment at 80 °C and 0 min (Figure 3B), the starch granules are deformed, and this deformation was also seen in the other treatments at 85 °C and boiling temperature, the gelatinization temperature is around 70 °C.

However, it should be mentioned that a longer time is required for the complete gelatinization of the starch. Therefore, gel and some coated granules can be seen in the treatments with 45 min of constant stirring (Figure 3C). This coating may be related to the surface layer or oleic acid coating of the starch granule, which could indicate the interaction of the main components of starch, in this case, amylopectin interacting with oleic acid on the surface of the granule (Figure 3D,E).

No whole grains were observed in the treatment at boiling temperature, due to the gelatinization of the starch by the thermal treatment, which leads to gel formation in which oleic acid molecules can immerse (Figure 3F). In this sense, the obtained images show a physical interaction between the starch component and the oleic acid. These results are consistent with the CI results (Figure 2), which showed a time-dependent increase, suggesting a homogeneous complex formation after a longer stirring time, in this case up to 45 min.

In addition, Figure 3 shows the waxy starch and ALC complex after the enzymatic attack and the possible fraction that can reach the colon. The untreated waxy starch was used as a control to study the effects of the amylopectin–oleic acid complexes (Figure 3H) on the morphology of the starch during enzymatic digestion. The micrographs reveal visible damage caused by the α-amylase and glucosidase enzymes in the waxy starch. The micrographs show clear pore formation in the granules indicating the enzymatic attack. However, despite this some lamellae structures that make up the starch granules and some starch granules remain.

When treated at 80 °C with stirring for 0 min (Figure 3I), the enzymatic damage is evident from the presence of pores on the surface of the granules and the lamellae as evidence of enzymatic attack, but when the stirring time is extended to 45 min (Figure 3J), the enzymatic damage becomes visible by the presence of large pores on the granule and the smooth surfaces, but the starch retains its structure, indicating resistance to enzymatic attack and associated with the coat seen in the same treatment before digestion. Nevertheless, it can be said that the damage is not severe and may be associated with partial gelatinization or protection from interaction with oleic acid.

In the treatment at 85 °C with 0 min of stirring (Figure 3K), after digestion, there is no presence of starch granules, and the remaining part has no pores, which can be associated with the gelatinization of the starch granules and consequently the formation of type 3 resistant starch; moreover, amylopectin–oleic acid complexes are also formed during the treatment. However, as can be seen above, when the stirring time was increased (85 °C for 45 min stirring), only some surfaces of the starch granules showed pores, and these micrographs demonstrate the resistance to enzymatic attack.

Finally, the micrographs of the treatments at boiling temperature and 0 min of stirring after the enzymatic attack show smooth surfaces and no whole granules. These micrographs demonstrate the resistance to the enzymatic attack, and the resistance can be related to the resistant starch type 5 through the amylopectin–oleic acid interaction. Above all, the treatment at 45 min of stirring shows a smooth surface without enzymatic damage, indicating the formation of RS, but a minimal part of the surface of the starch granules is damaged with pores, indicating that it is a starch resistant to enzymatic attack, but this treatment shows a greater resistance to enzymatic attack and this resistance may be associated with the presence of an amylopectin–oleic acid complex.

It is important to note that the amylopectin–oleic acid interactions were verified by DSC results, which showed that endotherms are associated with the presence of amylopectin–oleic acid complexes. The treatments conducted at 85 °C and boiling temperature with 45 min stirring had higher enthalpy than the other treatments and showed type I and II endotherms, indicating the most stable starch–lipid interactions. The results suggest that the formation of the amylopectin–oleic acid interaction induces enzymatic resistance.

These results show that the interaction between lipids and the major components of starch can be achieved by starch with high amylopectin content, representing a fast, simple, and inexpensive alternative to obtain these complexes. Consequently, amylopectin–lipid complexes obtained from waxy starch can be used to formulate a new ingredient for the food industry while maintaining the health benefits of resistant starch.

### 3.3. TEM Analysis

**Figure 4:**
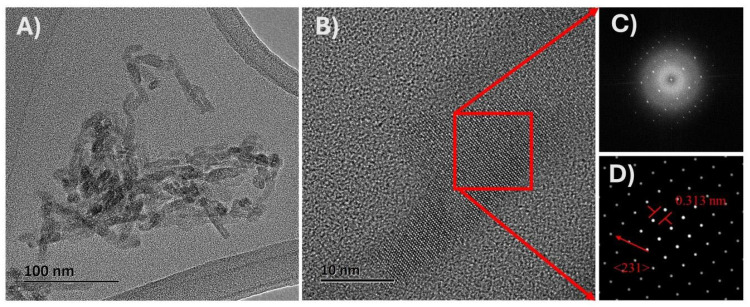
(**A**) Isolated nanocrystals, (**B**) red squares represent characteristic sections of the isolated nanocrystals, and (**C**) the representation of these sections in FT space and (**D**) the representation in inverse FT space.

Interpreting X-ray diffraction patterns for both modified and unmodified starches remains challenging, as it is not fully understood which components—such as fats, proteins, or crystalline structures—are affected by complexation. Starch functions as a composite material containing a range of components, with crystalline structures varying by source: cereals typically exhibit an orthorhombic crystal structure (type A), while tubers generally present a hexagonal one (type B). Each component, from fats to proteins to structural arrangements, contributes uniquely to the diffraction pattern [34]. Given these complexities, TEM analysis, which allows localized measurements within the starch granule, could offer critical insights by identifying which components and structures are specifically present and in what form. This capability could be key to clarifying how each constituent contributes to the overall structural and functional properties of the starch. Figure 4 shows the TEM images of isolated nanocrystals of native maize starch with high amylopectin content (waxy), showing isolated nanocrystals (100 nm scale). These structures consist of highly crystalline, elongated regions with an average crystallite size of 30 ± 6 nm in length, 10 ± 1 nm in width, and a notably thin profile (2–4 nm). Figure 4B shows a selected area with isolated, highly ordered nanocrystals around 10 nm in size. This specific area was used to determine the interplanar distance.

Figure 4C shows the investigated region in the nanocrystals where the FFT (Fast Fourier Transform) was applied using Digital Micrograph Software 3.5 to convert a signal in the spatial domain into its frequency domain representation. This can reveal information about the crystal structure of the sample, such as the orientation and the interplanar distance of the crystal. Figure 4D shows the results of applying a mask to the FFT. This makes it possible to focus on specific areas of an image, reduce noise, and obtain more meaningful and precise results that can be used to study crystal structures and features in detail according to the method proposed by Rojas-Molina et al. [35].

Figure 4D displays an interplanar distance of 0.313 nm along the <231> direction, confirming that maize starch with high amylopectin content (waxy) consists of nanocrystals with an orthorhombic structure. Comparing the distances obtained through TEM analysis with those reported by Rodriguez-Garcia et al. [36] for the orthorhombic crystal structure further supports this conclusion. These results confirm that the nanocrystals in this starch possess an orthorhombic structure, highlighting the importance of determining whether the starch modification proposed in this work influences this crystal arrangement.

### 3.4. Structural Analysis

**Figure 5:**
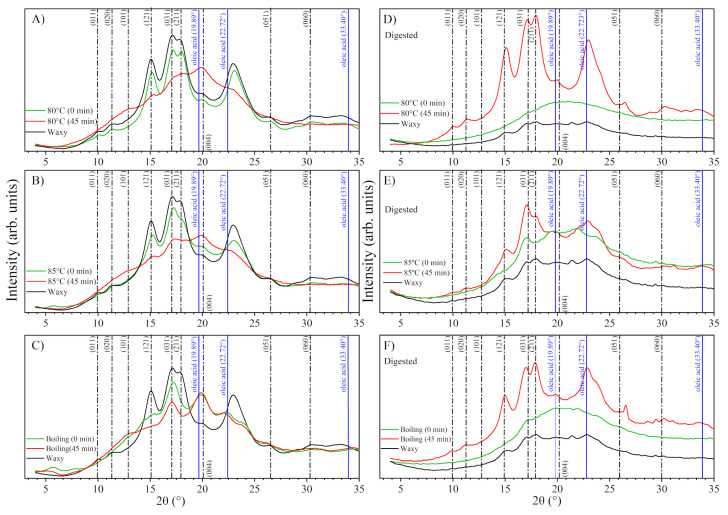
X-ray diffraction patterns for complexes formed with waxy starch and oleic acid at (**A**) 80 °C, (**B**) 85 °C, and (**C**) boiling temperature at different stirring times. Figure (**D**–**F**) show ALC treatments after digestion (RS fraction) at 80 °C, 85 °C, and boiling temperature, respectively, at different stirring times.

Figure 5 shows the X-ray diffraction patterns of the analyzed samples. In Figure 5A–C, the black line corresponds to the pattern obtained for untreated waxy starch, whereas the red and green lines correspond to the thermal treatments at different stirring times. In Figure 5D–F, the black lines correspond to the waxy starch after digestion without prior treatment, and the red and green lines correspond to the treated samples after digestion. The dashed lines indicate the positions of the indexed diffraction peaks for the starch. The diffraction pattern of untreated waxy starch matches the type A starch diffraction pattern, which corresponds to an orthorhombic crystal structure, as reported by Rodriguez-Garcia et al. [36]. The waxy starch diffraction pattern exhibits broad peaks and continuous halo, suggesting an amorphous phase; however, peak broadening may also result from crystallite size effects [43]. In this context, TEM analysis (Figure 4A) reveals that waxy starch contains highly ordered nanometer-scale structures (nanocrystals). These nanocrystals have not been previously reported or considered, and thus their role in forming complexes with amylose/amylopectin and lipids has not been accounted for.

At 0 min of stirring, the diffraction pattern retains features typical of type A starch, though with noticeably reduced intensity. As treatment temperature rises, prominent peaks associated with the (031) and (211) planes begin to broaden and overlap, suggesting partial disruption of the crystalline arrangement. This broadening indicates the onset of gelatinization (shown by the green lines in Figure 5A–C), where structural integrity starts to break down. However, the presence of distinct peaks reveals that a portion of the crystalline structure persists, likely due to insufficient hydrolysis to fully dissolve the ordered regions [44]. This retained crystallinity may influence subsequent interactions in complex formation with amylose and amylopectin. At the prolonged stirring times of 45 min, the diffraction pattern reveals a predominantly amorphous structure, characterized by an increased amorphous halo and a lack of distinct peaks. This effect is evident for all three treatment temperatures—80 °C, 85 °C, and boiling—as illustrated by the red lines in Figure 5A–C. The marked reduction in relative intensities indicates a significant loss of crystallinity. The combination of heat treatment and mechanical agitation disrupts starch crystallinity, leading to an increased degree of gelatinization. The diffraction patterns demonstrate that under these conditions, much of the original ordered structure breaks down, resulting in an amorphous matrix that lacks the defined peaks associated with a crystalline phase.

As previously mentioned, amylose and amylopectin can interact with lipids to form complexes that exhibit a characteristic V-type diffraction pattern. In this context, Sun et al. [41] investigated the effects of chain length and degree of unsaturation of the fatty acid on the structure, physicochemical properties, and in vitro digestibility of maize starch complexes. They found that these complexes produced a diffraction pattern with peaks at 7.5°, 13.5°, and 20.5° on the 2θ scale. Additionally, Marinopoulou et al. [30] identified crystalline complexes that exhibited characteristic peaks at 13.2° and 19.8°, indicating a V-type crystalline structure associated with the complexed amylose. Their findings highlight the presence of these peaks in the X-ray diffraction patterns of Hylon VII–fatty acid complexes, further reinforcing the notion of a distinct crystalline arrangement in these starch–lipid interactions [30]. However, the diffraction patterns reported by Marinopoulou et al. [30] correspond to partially gelatinized starch. This makes it challenging to distinguish between the peaks generated by the partially gelatinized starch and those produced by the formed complexes, particularly in samples treated at lower temperatures. Moreover, none of the authors indexed the V-type diffraction patterns, indicating that there are no associated crystallographic planes or Miller indices to explain the characteristic peaks observed in the diffraction pattern.

The diffraction patterns for treatments conducted at 80 °C and 85 °C, with stirring for 45 min, as well as for boiling temperatures across both stirring times, revealed a peak at 19.9° (Figure 5A–C). Although the peaks at 7.5° and 13.5° are not distinctly visible, this may be attributed to their overlap with the amorphous phase of the partially gelatinized starch. Given that the peak at 20.5° is associated with the V-type structure [2,30,45], the diffraction patterns for these treatments may suggest the formation of amylopectin–lipid complexes.

However, it is important to note that the previously mentioned crystalline structure has not been reported, nor has the V-type diffraction pattern been indexed. This suggests that the crystalline structure of the amylose–lipid complex does not form. In fact, the V-type structure was first proposed by Katz [46] to describe gelatinized starch, specifically amylose in a simple helical arrangement. Although the V-type pattern does not correspond to a new crystalline structure, the presence of peaks at 12° and 20° on the 2θ scale indicates that a short-range rearrangement occurs during the starch–lipid complexation. Therefore, the true structure of starch–lipid complexes remains an open question. Nevertheless, the results from the diffraction patterns in this study indicate that waxy starch requires a higher temperature (above 85 °C) and a longer stirring time to induce interaction between amylopectin and fatty acids, thereby facilitating the formation of amylopectin–lipid complexes.

Additionally, this study demonstrates that gelatinization causes amylose and amylopectin molecules to leach out, resulting in partially solvated nanocrystals with an orthorhombic structure. This process leads to an irreversible transition from ordered to disordered structures [33,34]. However, despite this transition, some crystal structures remain intact. It is important to note that even after boiling and stirring for 45 min, the starch is only partially gelatinized, as indicated in Figure 3N and Figure 5C. The partial gelatinization of starch during thermal treatment has also been reported by Rojas-Molina et al. [35]. This suggests that complex formation occurs only with gelatinized starch, particularly with those chains that have a wider molecular weight [37]. 

On the other hand, the diffraction patterns of the digested starch fractions indicate a conversion to a continuous amorphous halo for native waxy starch and samples treated with 0 min of stirring. According to Nieves-Hernández et al. [47], enzymatic activity does not cause significant alterations in starch crystallinity. Therefore, the changes observed in the diffraction patterns of waxy starch may be attributed to the method used to quantify resistant starch (RS), which involves thermal and chemical reactions. Additionally, gelatinization leads to the leaching of amylose and amylopectin and the partial solvation of nanocrystals [34]. As a result, samples that underwent no stirring treatment at low temperatures (80° and 85 °C) are more likely to exhibit this amorphous halo, reflecting a loss of atomic ordering in the system due to prior partial gelatinization.

However, the crystalline structure persists in all samples treated with 45 min of stirring, even after digestion. The diffraction patterns for these samples (Figure 5D–F) reveal strong, well-defined peaks at 15.08°, 17.12°, 17.86°, and 22.94° on the 2θ scale. These peaks correspond to an orthorhombic crystalline structure as reported by Rodriguez-Garcia et al. [36]. This phenomenon may be attributed to the formation of amylopectin–lipid complexes. After gelatinization, the formation of these complexes competes with the reorganization of starch molecules. Consequently, an inclusion complex can arise from the interaction of amylose or amylopectin with surrounding elements such as other starch molecules or fatty acids [48]. The presence of well-defined diffraction patterns in these treatments suggests that a network composed of gelatinized starch and amylopectin–lipid complexes forms around the partially gelatinized starch, effectively protecting the nanocrystals from enzymatic activity and thermal treatment. According to this theory, it is the amylopectin–oleic acid complex and the highly compacted crystal structures (nanocrystals of pyroglucans) that reach the large intestine as the resistant fraction. Furthermore, these nanocrystals are part of RS2, as suggested by Rojas-Molina et al. [35].

**Figure 6:**
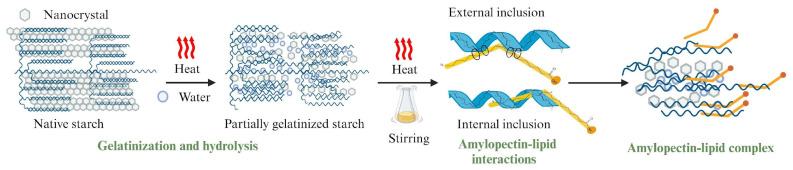
Proposed kinetic model for the formation of the amylopectin–lipid complex.

However, additional studies are necessary to validate this theory. Then, based on SEM measurements and diffraction pattern data, the proposed kinetic model for amylopectin–lipid complex formation is shown in Figure 6. Initially, partial gelatinization of the starch occurs. Following this, the fatty acid is added, and with mechanical stirring, the fatty acid chain interacts with the gelatinized starch fraction. The fatty acid can then position itself either on the outer or inner regions of the amylopectin chain, forming the complex. Thus, the resistant starch fraction formed by partially gelatinized starch consists of a combination of pyroglucan nanocrystals and amylopectin–oleic acid complexes.

### 3.5. Thermal Properties

**Figure 7:**
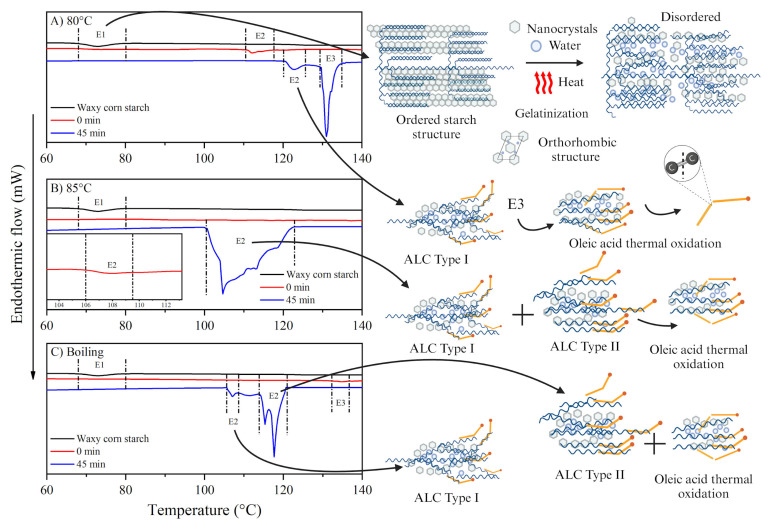
Thermograms of amylopectin–lipid complexes at different temperatures (**A**) 80 °C, (**B**) 85 °C, and (**C**) boiling temperature, with 0 and 45 min of stirring. * ALC—Amylopectin–lipid complex.

Depending on their structural arrangement, two types of starch–lipid interactions are considered: type I and II. Since each of these interactions have been described as a function of their endothermic transition with respect to the corresponding amylose–lipid interaction, these complexes can be studied by DSC, and therefore classified as type I at 90–110 °C (endotherm 1) and type II at 110–130 °C (endotherm 2) [9]. Figure 7 shows the thermal profiles of waxy starch, and the complex formed after thermal treatment at different stirring times. DSC analysis was used to determine the interactions between the amylopectin molecule and the oleic acid, and the following endotherms were found: E1: Starch gelatinization temperature due in part to solvation of the orthorhombic nanocrystals [9]; E2: Amylopectin–oleic acid complex type I; and E3: Amylopectin–oleic acid complex type II and thermal oxidation of oleic acid [49,50].

The formation of amylopectin–oleic acid complexes depends on the method used and the set temperature. However, the necessary conditions for this complex formation still need to be clarified [51]. Waxy starch without treatment has a gelatinization temperature from 58.6 to 76.3 °C with an enthalpy of 7.13 J/g. This transition corresponds to the gelatinization of waxy starch (E1). The shift in gelatinization transition (69.53 to 81.1 °C) was observed when the treatment was carried out at 80 °C and stirred for 0 min. However, the enthalpy decreased (7.14 to 1.13 J/g), indicating that partial gelatinization of the starch occurred, resulting in the release of amylopectin. The treatments at 85 °C and 0 min stirring time showed the presence of a second endotherm (E2), ranging from 106.26 to 108.72 °C, and the treatment at boiling temperature with 0 min stirring time showed three endotherms: E1 (68 to 76 °C, ΔH: 0.54 J/g), E2, and E3.

The presence of E1 in the treatments indicates the presence of residual non-gelatinized starch. While the presence of E2 corresponds to the formation of type I amylopectin–oleic acid complexes. However, the left shift of this endotherm indicates the presence of type II amylopectin–oleic acid complexes. In addition, endotherm 3 (E3) could indicate the thermal oxidation of oleic acid. The formation of type I complexes could be related to temperature, as heat treatments above the gelatinization temperature and constant stirring can promote solvation of the nanocrystals and leaching of the amylopectin chains [33,34], allowing the helices in the amylopectin branches to interact with the oleic acid.

On the other hand, the stirring time can promote the debranching/hydrolysis of amylopectin, releasing smaller molecules (dextrin) that can interact more efficiently with oleic acid [9,52] and consequently, form complexes with greater thermal stability, as shown by the treatments with longer stirring time and higher temperature.

The treatment at boiling temperature and 45 min of stirring showed the formation of type II amylopectin–oleic acid complexes with the highest enthalpy (13.02 J/g). These results demonstrate the existence of type II amylopectin–oleic acid complexes and show the thermal stability of the interaction. The endothermic E2 transition was observed at 80 and 85 °C and 0 min of stirring, while the 100–122 °C transition at the 85 °C treatment with 45 min of stirring was undefined and can be explained by a mixture of type I and II complexes. A third transition (E3) can be seen in the thermograms. This transition is undefined and is observed at 110 and 130 °C. On the other hand, the thermal oxidation reactions of free oleic acid involve the cleavage of the C=C double bond on carbon 9 of the aliphatic chain at 120–140 °C. Therefore, this transition can be associated with the presence of an amylopectin–lipid complex, the possible thermal oxidation of oleic acid (E3) and, at the same time, a dissociation of oleic acid-amylopectin complexes in the range of 100–122 °C [49,50].

The results obtained for the thermal properties show that the formation of amylopectin–oleic acid complexes increases at temperatures above 80 °C, and that the interaction time influences the stability of the complex formed. This behavior is related to the CI results, which show that the strongest interaction occurs at 85 °C and at the boiling temperature, both at 45 min of stirring. Furthermore, the thermal properties confirmed the interaction between amylopectin and oleic acid and emphasized the higher stability of the complexes at boiling temperature and 45 min of stirring than in the other treatments used in this study.

### 3.6. Fourier Transform Infrared Spectroscopy (FTIR)

**Figure 8:**
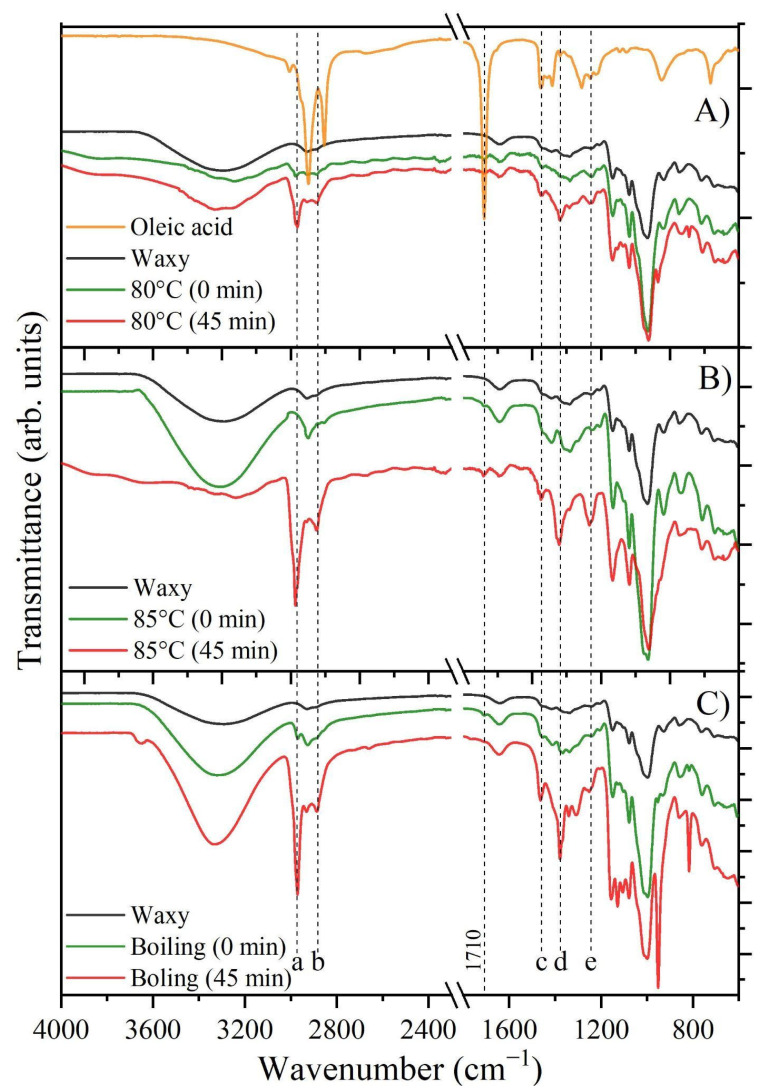
FTIR-ATR spectra for complexes formed from oleic acid and waxy starch at different temperatures (**A**) 80 °C, (**B**) 85 °C, and (**C**) boiling temperature. Different letters (a, b, c, d, and e) correspond to the main bands associated with amylopectin–lipid complexes formation.

Figure 8A–C shows the infrared spectra of oleic acid, untreated waxy starch, and the amylopectin–lipid complexes formed from waxy corn starch at 80, 85 °C, and boiling temperature at 0 and 45 min of stirring. Figure 8A displays the infrared spectrum of oleic acid and untreated waxy starch. These spectra correspond to the characteristic fingerprints of these materials. The spectra of the treated samples exhibit the characteristic bands associated with untreated starch. However, several new bands appear at 2970, 2888, 1464, 1379, and 1251 cm^−1^. These additional bands may indicate modifications in the molecular structure of the starch, suggesting potential interactions or chemical changes resulting from the treatment process. The bands at 2970 cm^−1^ (a) and 2888 cm^−1^ (b) correspond to the stretching of C–H bonds in the methyl (–CH₃) and methylene (–CH₂) groups of oleic acid [2,26,53]. It is important to note that these bands appear shifted to higher wavenumbers compared to the typical position of oleic acid, suggesting that there is an interaction between the polymeric matrix and the fatty acid chains via these functional groups. This implies that it is not a simple mixture but rather an interaction-driven complex formation. Similarly, the 1464 cm⁻^1^ (c) band, related to C–H bending vibrations in methylene groups, and the 1379 cm⁻^1^ (d) band, associated with CH₃ bending, appear shifted to lower wavenumbers and increase in intensity with higher thermal treatment and stirring time. This further supports the hypothesis of a chemical interaction and confirms the formation of a complex between the starch and fatty acid. The 1240 cm⁻^1^ (e) band, tied to C–O stretching, indicates potential hydrogen bonding or esterification between hydroxyl groups in starch and oleic acid, enhancing structural stability [6,52]. Finally, the intense band at 1710 cm⁻^1^, characteristic of oleic acid, was almost absent in the spectrum of the treated samples. This absence is likely due to the washing step applied to treated samples to remove any free oleic acid residue from the mixture. Therefore, studies reporting this band as an indicator of complexation may, in fact, be detecting residual free oleic acid in the system. In this context, the lack of intensity in this band serves as further evidence of complex formation [54], a phenomenon similarly observed in other complex systems, such as oleic acid with nanoparticles and chitosan, where the normal mode also disappears [55].

Furthermore, as the temperature and stirring time increase, the intensity of these bands also rises, suggesting a greater degree of complexation. Thus, it can be said that the formation of complexes is favored by longer stirring times at 85 °C and at boiling temperature.

### 3.7. Rapidly Digested Starch, Slowly Digested Starch, and Resistant Starch Quantification

Table 1 shows the percentage of rapidly digestible starch (RDS), slowly digestible starch (SDS), and resistant starch (RS) formed from the amylopectin–lipid complexes and the waxy starch. The quantification of the different digestible starch fractions shows significant differences in the RDS content between the treatments at 80 and 85 °C and the treatments at boiling temperature and at different stirring times (0 and 45 min). The highest content of rapidly digestible starch (90.07 ± 1.1%) was observed in the treatment with 0 min of stirring. However, no differences (*p* > 0.05) in SDS content were observed between the treatments.

The increase in RS content was related to the increase in treatment temperature and stirring time. However, no significant differences were found between the treatments at 80 and 85 °C and 45 min stirring time (27.74 ± 0.23 and 26.61 ± 0.45%, respectively). On the other hand, the treatment at boiling temperature and 45 min of stirring time was statistically different from the other treatments as it had the highest amount of RS (49.33 ± 1.61%).

The RS values at 80 °C and 0 min of stirring were lower than the other treatments because gelatinization of waxy starch occurs in the range of 68 to 75 °C. Therefore, the treatment at 80 °C resulted in the gelatinization of starch, while the stirring time of 0 min does not show an effect on the formation of RS. Although an amylopectin–oleic acid interaction was observed in the CI and DSC results, this interaction between amylopectin and oleic acid may help to resist the digestion process of starch because the amylopectin–oleic acid complex restricts the access of enzymes so that the complexes are resistant to digestion [56,57].

Meanwhile, the effect of temperature on RS content was not significant in the 80 and 85 °C treatments, but a significant difference was observed in the boiling temperature treatments. Interestingly, the samples that were stirred for 45 min were significantly different from the samples that were stirred for 0 min, suggesting that stirring time has a greater effect on RS formation than temperature.

It has previously been shown that waxy starch has a low ability to interact with fatty acids due to the length of its branches. These branches represent a steric hindrance to interaction with fatty acid chains [58]. However, the results of this study suggest that the effect of temperature on RS content is due to thermally mediated gelatinization of the starch, which leads to leaching of the amylopectin chains (which are fragmented with constant stirring to form further complexes with the oleic acid). In this sense, the formation of amylopectin–oleic acid complexes is reflected in the DSC results (Figure 7), especially in the endotherms observed for the type I and type II complexes. Remarkably, the treatment at boiling temperature and a stirring time of 45 min showed an enthalpy of 13.02 J/g in the DSC results, indicating that it is a stable complex. In agreement with the RS results, the treatment at boiling temperature and 45 min of stirring time showed the highest resistance to digestion among all treatments. The observed effect may be related to the fact that type I amylopectin–oleic acid–lipid complexes can dissociate after boiling, suggesting that the resistance to digestion in boiling temperature treatments may originate from type II amylopectin–oleic acid complexes [56,57]. Finally, waxy starch showed a 1.3-fold increase in RS at boiling temperature and 45 min stirring time compared to untreated starch, confirming an increasing formation of amylopectin–oleic acid complexes.

Therefore, CI is associated with interaction time and increase in RS (r = 0.86, *p* < 0.05), in contrast, RDS is negatively associated with stirring time (r = −0.82, *p* < 0.05). The increase in RS is associated with CI (r = 0.85, *p* < 0.05), indicating the formation of an interaction between amylopectin and oleic acid. From the above results, it can be concluded that at a controlled temperature and constant stirring time, the formation of amylopectin–oleic acid complexes is possible, and these complexes lead to an increase in RS5.

## 4. Conclusions

Waxy starch, which is high in amylopectin, can form complexes with oleic acid either through self-assembly or by positioning itself along the outer regions of the amylopectin chain. This occurs under controlled stirring times and at temperatures above 85 °C. The structural characteristics of these complexes were identified by the formation of V-type diffraction patterns. For native waxy starch, both X-ray XRD and TEM revealed an orthorhombic crystalline structure, which experiences partial degradation during the gelatinization process.

The formation of the amylopectin–oleic acid complex occurs as soon as the starch gelatinizes, because during gelatinization, the starch granules swell and break up, releasing amylopectin molecules. Simultaneously, the constant stirring plays a decisive role in the modification of the amylopectin chains. This hydrolysis can be induced by constant mechanical agitation.

X-ray diffraction patterns and TEM images revealed the presence of nanocrystals with orthorhombic crystal structure in waxy starch, which form part of RS5 for partially gelatinized starch. According to the XRD analysis of the resistant fraction of ALC, the presence of well-defined diffraction patterns after digestion suggests that for partially gelatinized starch, a network composed of gelatinized starch and amylopectin–lipid complexes protects the nanocrystals from enzymatic activity and thermal treatment. Thus, the total resistant fraction is the sum of the pyroglucan nanocrystals (RS_2_) and the amylopectin–oleic acid complex, as well as some whole grains that resist all treatments. Finally, these results have shown that the formation of starch–lipid complexes can be achieved with starch with high amylopectin content and represents a fast, simple alternative.

## Figures and Tables

**Table 1:** Content of rapidly digested starch (RDS), slowly digested starch (SDS), and resistant starch (RS) in the different treatments for the formation of amylopectin–lipid complexes with waxy corn starch.

Treatment		RDS (%)	SDS (%)	RS (%)
Temperature (°C)	Time (min)			
Native		61.50 ± 0.51	3.51 ± 0.22	20.83 ± 0.3
80	0	90.07 ± 1.10 ^a^	2.31 ± 1.63 ^d^	6.4 ± 0.74 ^d^
	45	35.81 ± 0.10 ^d^	31.87 ± 1.22 ^a^	27.74 ± 0.23 ^b^
85	0	79.98 ± 2.70 ^b^	6.89 ± 0.18 ^d^	12.63 ± 0.39 ^c^
	45	44.26 ± 3.20 ^c^	21.24 ± 0.51 ^c^	26.61 ± 0.45 ^b^
Boiling	0	50.39 ± 2.1 ^c^	32.90 ± 0.31 ^a^	11.05 ± 1.90 ^c^
	45	24.56 ± 1.2 ^e^	25.61 ± 1.30 ^b^	49.33 ± 1.61 ^a^

The mean ± standard deviation of two replicates is shown. Different letters in the same column are statistically different (*p* < 0.05).

## Data Availability

The original contributions presented in the study are included in the article, further inquiries can be directed to the corresponding author.

## Appendix A

**Table A1:** Results of Tukey’s multiple comparisons test, showing the mean differences, 95% confidence intervals, and adjusted *p*-values for the comparisons between temperature treatments (80 °C, 85 °C, and boiling). Statistically significant differences are indicated by asterisks, with **** representing *p* < 0.0001 and ** representing *p* < 0.01.

Tukey’s Multiple Comparisons Test	Mean Diff.	95.00% CI of Diff.	Adjusted *p*-Value	
85 °C vs. 80 °C	3.978	2.475 to 5.481	****	<0.0001
Boiling vs. 80 °C	5.963	4.460 to 7.466	****	<0.0001
Boiling vs. 85 °C	1.985	0.4820 to 3.488	**	0.0073
